# Supplementary figures and images for: Prevalence of permethrin-resistant kdr mutation in head lice (Pediculus humanus capitis) from elementary school students in Jeddah, Saudi Arabia
Source: PeerJ. 2023 Oct 25;11:e16273. doi: 10.7717/peerj.16273 (PMC10612490; doi:10.7717/peerj.16273)

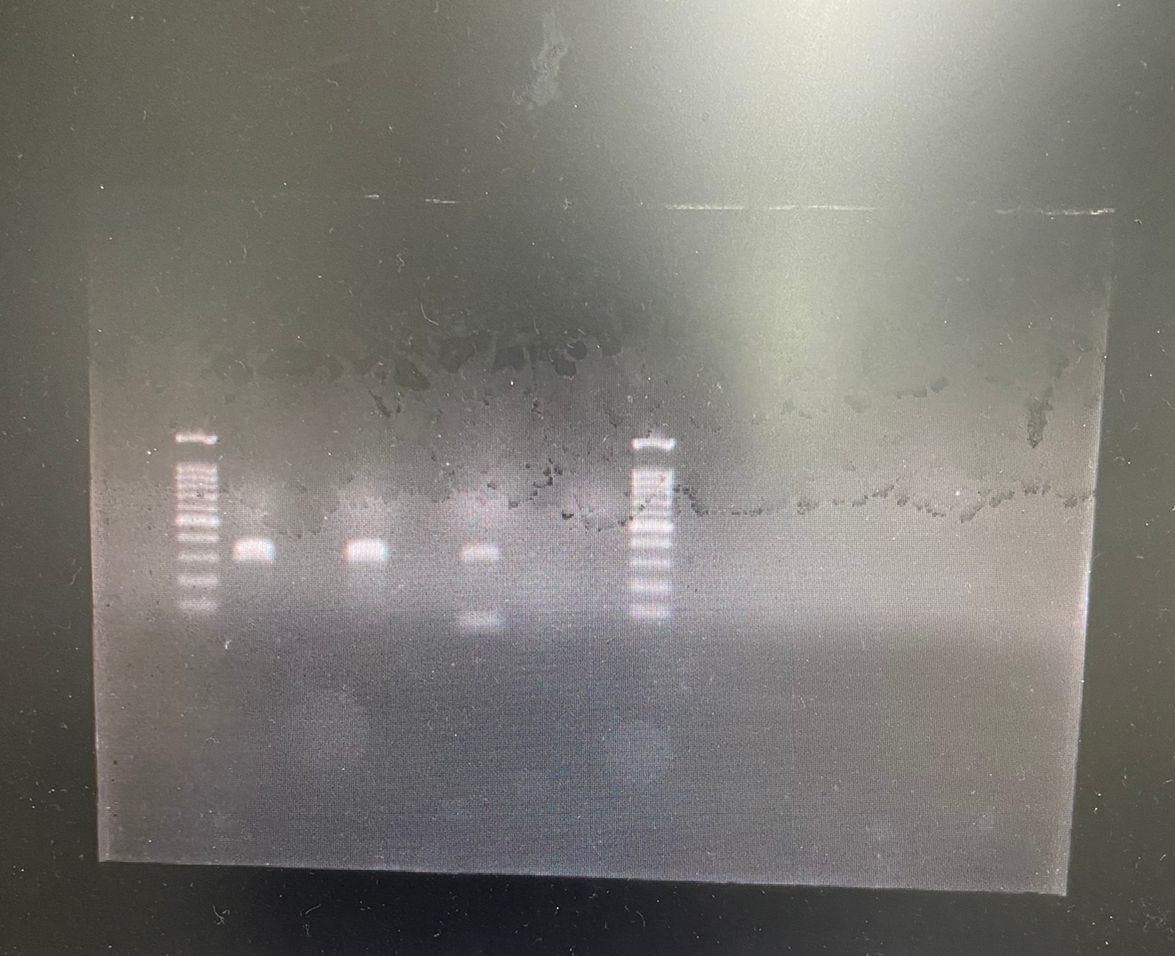

Supplement: Supplemental Information 1 [file peerj-11-16273-s001.jpg]
